# High temperatures during microsporogenesis fatally shorten pollen lifespan

**DOI:** 10.1007/s00497-021-00425-0

**Published:** 2021-07-07

**Authors:** Maurizio Iovane, Giovanna Aronne

**Affiliations:** Department of Agricultural Sciences, University of Naples Federico II, Naples, Italy

**Keywords:** Microgametophyte, Pollen viability, Pollen germination, Heat waves, Pollen development, Tomato

## Abstract

Many crop species are cultivated to produce seeds and/or fruits and therefore need reproductive success to occur. Previous studies proved that high temperature on mature pollen at anther dehiscence reduce viability and germinability therefore decreasing crop productivity. We hypothesized that high temperature might affect pollen functionality even if the heat treatment is exerted only during the microsporogenesis. Experimental data on *Solanum lycopersicum* ‘Micro-Tom’ confirmed our hypothesis. Microsporogenesis successfully occurred at both high (30 °C) and optimal (22 °C) temperature. After the anthesis, viability and germinability of the pollen developed at optimal temperature gradually decreased and the reduction was slightly higher when pollen was incubated at 30 °C. Conversely, temperature effect was eagerly enhanced in pollen developed at high temperature. In this case, a drastic reduction of viability and a drop-off to zero of germinability occurred not only when pollen was incubated at 30 °C but also at 22 °C. Further ontogenetic analyses disclosed that high temperature significantly speeded-up the microsporogenesis and the early microgametogenesis (from vacuolated stage to bi-cellular pollen); therefore, gametophytes result already senescent at flower anthesis. Our work contributes to unravel the effects of heat stress on pollen revealing that high temperature conditions during microsporogenesis prime a fatal shortening of the male gametophyte lifespan.

## Introduction

Successful interaction of pollen with the environment is essential in agricultural systems because most crop species are cultivated to produce seeds and/or fruits and therefore need fertilization to occur. Considering that in higher plants each pollen grain is a haploid gametophyte of only two (max three) cells generated by the diploid sporophyte through a meiosis (microsporogenesis), pollen ephemeral life is limited at producing two gametes (microgametogenesis), possibly after being transferred to a plant different from the one that generated it (cross-pollination). Despite the smallness of its size and the shortness of its life pollen grains of any species are autonomous organisms, genetically different from any other and subject to the interaction with the environment. Pollen grains are exposed to natural selection and can influence the genetic constitution of the resulting sporophytic generation (Mulcahy 1979). The possibility to screen the individuals best adapted to specific environmental conditions is the concept which gametophytic selection is based on and proposed as alternative to the much wider used sporophytic selection for crop species (Hormaza and Herrero 1996). Although the studies on the effects of external factors on pollen biology are mainly focused on mature and/or germinated grains, environmental conditions affect pollen at all development stages, including the microsporogenesis (Pacini and Dolferus 2019).

Interaction of pollen with environmental factors is relevant not only for crop production on Earth but also for long term mission in Space. Indeed, pollen functionality has a key role in implementing bioregenerative life support systems (BLSSs) for long duration space-flight missions. Therefore, research on interaction between pollen and environmental factors, in addition to space factors as simulated microgravity (De Micco et al. 2006), is essential to realize the seed-to-seed cycle also on board spacecraft.

Considering recent projections of climate changes, a rising concern for gradual increases and abrupt variations of temperature is leading research to select genotypes tolerant to high temperatures or heat waves and to clarify the physiological processes involved in heat-stress responses (Hedhly et al. 2009; Mesihovic et al. 2016). Generally in plants, pollen functionality is strongly affected by high temperatures, alone or in interaction with RH (Aronne 1999). More particularly, high temperatures are well known to affect pollen viability, pollen germinability and fertilization both in crops and in other plant species (Pérez et al. 2019; Aronne et al. 2015).

Pollen sterility induced by high temperature stress is an agricultural problem because it limits the productivity of many crop species (Pacini and Dolferus 2019). Negative effects of high temperature on pollen functionality constitute possible bottlenecks in plant life cycle of many species and conditions (De Micco et al. 2014; Aronne 2017). Therefore, a deep understanding on how high temperatures affect pollen functionality is essential to ensure food security.

At present, a special need to deepen pollen reaction to altered temperature conditions is relevant not only to predict the effect of gradual global warming on crop productions, but also to prevent the effect of sudden changes of temperatures during single producing cycles. Such events of heat waves are not rare in open field and in greenhouses and might strongly affect quantity and quality of crop production (Ledesma and Kawabata 2016).

Previous studies on the effect of high temperature at flowering, generally focus their attention on the functionality of the male gametophytes (Aronne et al. 2006, 2021; Djanaguiraman et al. 2019) and are performed incubating mature pollen grains samples after their release from the anthers (Dafni 1992). However, negative effects of high temperature before pollen development have been described on microsporogenesis of several species (Porch and Jahn 2001; Szalay et al. 2019; Masoomi-Aladizgeh et al. 2020). Among crops, a considerable amount of studies has been conducted to assess heat stress on tomato pollen, because of its agronomic relevance and its exposure to temperature fluctuations during flowering, both in open field and in greenhouse (Firon et al. 2006; Paupière et al. 2017a). In tomato heat stress is also reported to interfere with tapetum formation, exine deposition and vacuolization of mature microspores (Giorno et al. 2013) and also with starch accumulation (Pressman et al. 2002). These heat stress-induced defects are produced during the microsporogenesis, may strongly affect the progression of male gametogenesis, and therefore the correct formation and functionality of mature pollen. Notwithstanding the general knowledge on the effect of temperature on plant reproduction, specific studies aimed to investigate on possible relations between a heat stress occurred during the microsporogenesis and a later effect on the gametogenesis have been generally neglected.

Tomato is frequently used as model species for studies on interaction between high temperature and reproductive biology (Paupière et al. 2017b; Pham et al. 2020). In this work, we grew dwarf tomato plants under finely controlled temperature conditions to deepen the timing of pollen ontogenesis and to test the hypothesis that high temperature occurring during microsporogenesis can affect pollen formation and especially subsequent functionality of the male gametophyte.

## Material and methods

The experiment was performed in two equal growth chambers (VELP^®^, FOC 200IL). We used plants of *Solanum lycopersicum* ‘Micro-Tom’, a dwarf tomato selected to perform experiments in space. It is considered as an ideal model plant for experiments in confined environment because of its small habitus and its short life cycle (Matsukura et al. 2008). Among tomato cultivars, Micro-tom is used to elucidate the molecular mechanisms of reproductive biology traits in tomato because in addition to the small size and the short life cycle, it can be easily crossed with other tomato cultivars and genetically transformed (Sun et al., 2006). Moreover, the whole genome of Micro-Tom has been sequenced and made available in the ‘TOMATOMICS’ database (Kobayashi et al., 2014).

Plants were grown in plastic pots on a 1:1 (v/v) soil and perlite medium. A photosynthetic photon flux (PPF) of 200 μmol m^−2^ s^−1^ with a 16 h d^−1^ photoperiod was provided by white LEDs and relative humidity was kept at 70% ± 5% throughout the whole experiment. At first, we grew 18 plants of Micro-Tom in a single growth chamber with an air temperature of 22 °C ± 0.5 °C (control temperature) generally considered as optimal for tomato (Sato et al. 2001; Matsuda et al. 2014). As soon as the primordia of the first inflorescences became visually distinguishable, we moved half plants in the other growth chamber in which all parameters were the same but the air temperature of 30°C ± 0.5°C (high temperature). This approach was adopted to avoid possible effects of the two temperatures on vegetative phase of the plant growth and to ensure that the plants were exposed at the two different temperatures only during microsporogenesis. The high temperature treatment was designed and set in the growth chamber according to the screening for pollen tolerance in tomato reported by Paupière et al. (2017a) in which maximum average day temperatures for heat-tolerant tomato genotypes originated from location with hot environmental conditions varies from 28°C to 31°C.

**Fig. 1:**
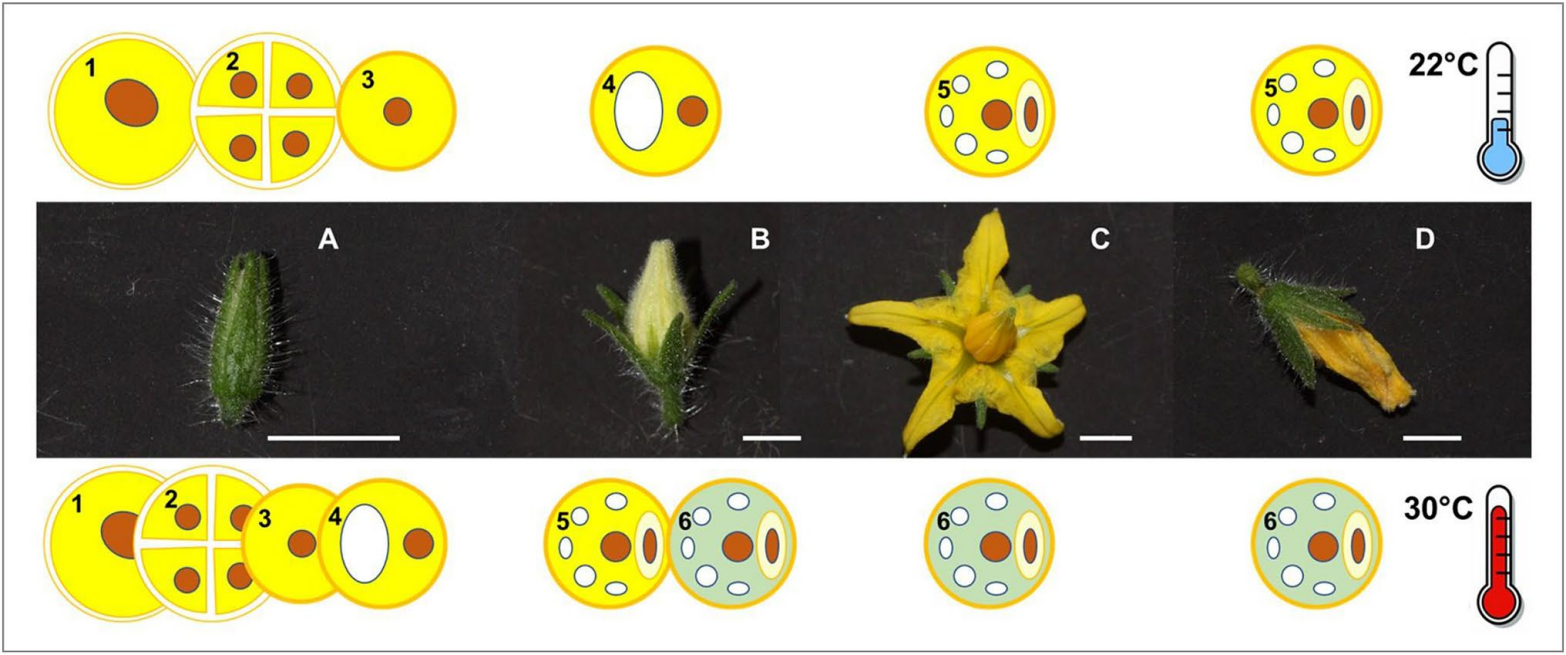
Flowering stages and relative pollen development phases in flowers of tomato plants grown at 22 °C and 30 °C. Flowering stages: **a** Closed calix, **b** Closed corolla, **c** Anthesis, **d** Post-anthesis. Pollen developmental phases: (1) Pollen mother-cell, (2) Tetrad, (3) Uni-cellular microspore, (4) Vacuolated stage, (5) Bi-cellular pollen grain, (6) Dead pollen grain. Bars = 2 mm

We evaluated pollen functionality on a total amount of 72 flowers for each temperature treatment. For every single flower, the pollen from each anther was used for a single temperature treatment as specified below. To track pollen abortion, pollen viability and germinability during microsporogenesis and gametogenesis, therefore throughout flower lifespan, we collected flowers at four different flowering stages: (a) Closed calix, (b) Closed corolla, (c) Anthesis, (d) Post-anthesis (Fig. 1).

Pollen abortion at the end of microsporogenesis was measured using lactophenol-cotton blue stain that detects the presence of cytoplasm in pollen grains (Rodriguez-Riano and Dafni 2000). Pollen abortion was tested on 18 flowers for each temperature treatment occurring during microsporogenesis. Therefore, the pollen samples were collected from 36 anthers of different flowers buds with closed corolla. At this stage, we were able to compare the effect of the two temperature treatments occurring during microsporogenesis on pollen abortion, after microsporogenesis had ended. Each pollen sample was obtained by gently opening the undehisced anther with tweezers and spreading the pollen from a single anther directly on a 1 µL droplet of water previously placed on a slide. One droplet of 1µL of Lactophenol-cotton blue stain was added on each pollen sample and slides were mounted with a cover slip. We scored as non-aborted the pollen grains stained dark/blue and as aborted the ones that remained faint/colourless. Abortion percentage was measured counting at least 200 pollen grains per slide for a total of 36 slides (18 flowers × 2 temperature treatments). Scoring was made using an Olympus^®^ BX-60 light microscope.

To compare the effect of the two temperatures during microsporogenesis on gametophyte functionality, we evaluated pollen viability and germinability after 72 hours of incubation at 22 °C and 30 °C. Half of the flowers developed under 22 °C were incubated at 22 °C and the other at 30 °C. Similarly, half of the flowers developed under 30 °C were incubated at 22 °C and the other at 30 °C. Overall, for each temperature treatment we incubated 27 flowers at 22 °C and 27 flowers at 30 °C. More specifically, incubated flowers were distinct into three categories corresponding to the stages of (1) closed corolla, (2) anthesis, (3) post anthesis. Each flower of the same phenological stage was excised from a single inflorescence, placed in open Petri dishes and incubated in two different growth chambers set at 22 °C and 30 °C with 70% RH.

To minimize possible inter-flower variations, 4 anthers per flower/bud were each used to assess: (1) pollen viability after 72 h incubation at 22 °C, (2) pollen viability after 72 h incubation at 30 °C, *3) pollen germinability after 72 h incubation at 22 °C, (4) pollen germinability after 72 h incubation at 30 °C.

Both for pollen viability and in vitro germination, three pollen samples of at least 200 grains were analysed per anther and scored with an Olympus^®^ BX-60 light microscope.

To test pollen viability, we used diaminobenzidine (DAB) reaction because of its ease of use and its reliability to distinguish between viable and unviable pollen (Rodriguez-riano and Dafni 2000). For each sample, pollen from a single anther was spread on a slide. 1 µL droplet of DAB solution was added on each pollen sample and slides were gently warmed on a heating plate. When pollen was dry, slides were mounted with a cover slip and immediately microscope analysed. We scored as viable only pollen grains that turned totally black or dark brown.

In vitro pollen germination was tested through the hanging drop method using a germination medium modified from Karapanos et al. (2010) and Song et al. (1999) and optimized for Micro-Tom pollen. One droplet of germination medium was deposited on a cover slip and pollen from a single anther was released inside the droplet before turning the cover slip upside down. To prevent dehydration, slides with the hanging drops were stored in petri dishes sealed with parafilm. To evaluate in vitro pollen germination, hanging drops were incubated at either 22 °C or 30 °C for 24 h. Grains were scored as germinated when the pollen tube was longer than the pollen diameter.

To analyse pollen ontogenesis, we collected flower buds at different developmental stages, extracted the anthers with the help of an Olympus^®^ SZX10 stereomicroscope and squashed them on microscope slides. We used different staining techniques on different anthers of single flowers. More specifically, to distinguish between well developed and aborted pollen we used aceto-carmine, toluidine blue and aniline blue staining procedures (Dafni 1992). Stained samples were observed with an Olympus^®^ BX-60 light and epi-fluorescent microscope and results were cross-linked to get a comprehensive overview of the processes.

Data were analysed with IBM^®^ SPSS Statistics. The Shapiro Wilk’s test and Levene's test were, respectively, used to assess the normality and homogeneity of the variances of the datasets. Differences between the two temperatures during microsporogenesis on pollen abortion were compared with a t-test (*p* < 0.05). As concern pollen viability and germination, results were obtained from a three-factorial design (2 temperatures during microsporogenesis, 2 of incubation, 3 flower/bud types, 9 replicates) and significant differences were tested with a three-way ANOVA (*p* < 0.05). The Tukey HSD test (*p* < 0.05) was used for post-hoc analysis. Results expressed as percentage were converted with arcsine function.

## Results

The Micro-tom plants grown at 22 °C during the vegetative phase and subsequently grown at 22 °C and 30 °C during the reproductive phase, compared to those always kept at 22°, did not show visual differences in terms of growth rate, flowering time and flower morphology (colour and size).

Microscopic analysis showed that the microsporogenesis occurred in buds at the stage of closed calix and is completed at the stage of closed corolla both in flowers developed at 22 °C and in those developed at 30 °C.

The lactophenol-cotton blue test performed on pollen coming from flower buds with closed corolla revealed that the number of not aborted/well-formed pollen grains was high in flowers developed under both temperature treatments. However, at closed corolla stage, percentage of not aborted pollen resulted only slightly lower (*p* < 0.05) at 30 °C (97.64%) compared to 22 °C (99.22%). Nevertheless, higher temperature during microsporogenesis did not lowered so much the number of well-formed pollen grains. Overall, the lactophenol-cotton blue test proved that higher temperature during microsporogenesis did not interfere so much with the first stages of pollen development and that the great majority of the grains in the anthers at the stage of closed corolla was well formed and functional in both temperature treatments.

**Fig. 2:**
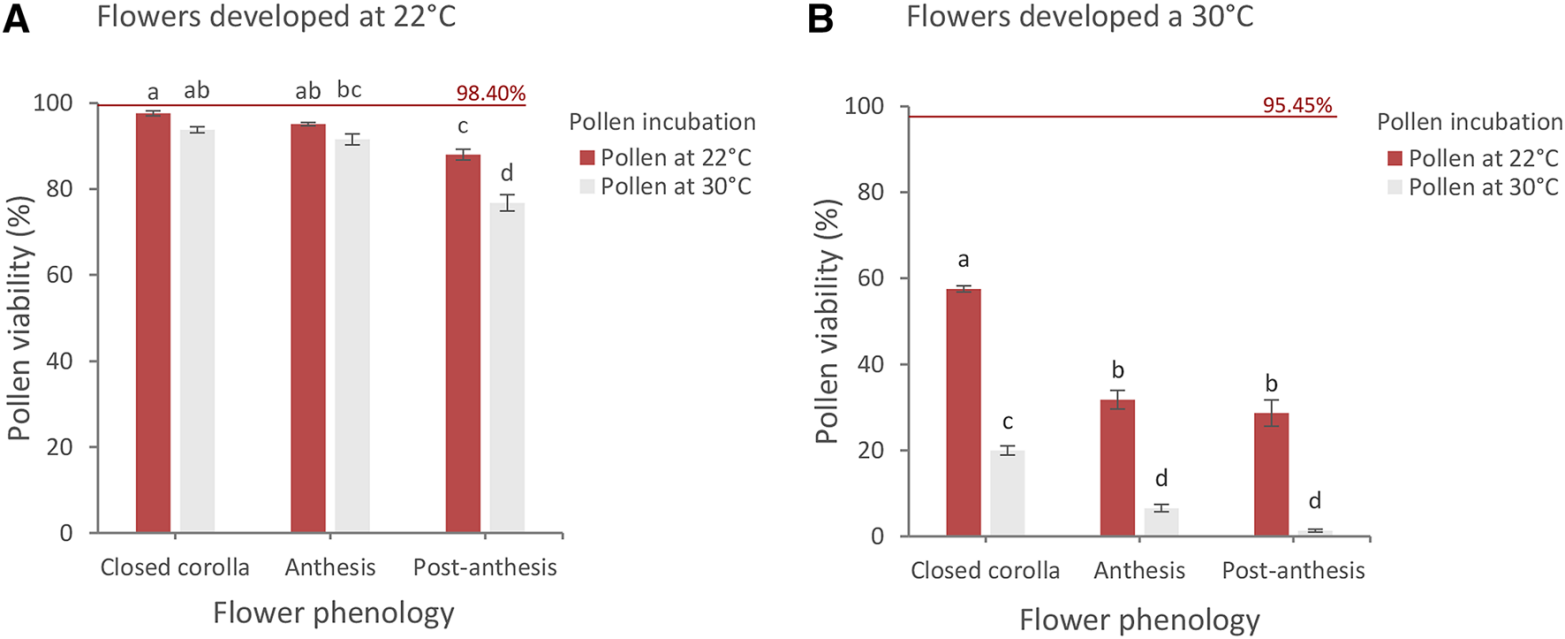
Viability of pollen from flower at different phenological stages and incubated for 72 h at 22 °C and 30 °C. Data from flowers in which microsporogenesis occurred at 22 °C **(a)** and 30 °C **(b)**. Horizontal lines show well-developed pollen percentage at anthesis before incubation. Significant differences between phenological stages are expressed with different letters (*p* < 0.05). Bars represent ± SE

Prolonging the temperature treatments on flowers up to the stage of anthesis and testing pollen viability with the DAB enzymatic reaction, we verified that most of the grains at anther dehiscence resulted viable in flowers from both temperature treatments. Percentage of viable grains in flowers developed at 22 °C was statistically not different to that of flowers at 30 °C (98.40% and 95.45% respectively, *p* < 0.01) (Fig. 2). Therefore, high temperature during flower development and microsporogenesis did not lowered pollen viability at the beginning of the anthesis.

The scenario changed completely when we collected the pollen from plants treated at the two temperatures during flowering/microsporogenesis development and subsequently incubated the grains at the same and cross changed two temperatures. Results of the DAB viability tests were analysed according to a three-factorial design including 2 temperatures during microsporogenesis, 2 incubation temperatures and 3 flower/bud types. The three-ways ANOVA revealed that the temperature during microsporogenesis resulted the main factor affecting pollen viability (F = 6455.24, *p* < 0.05) along all the flowering stages: closed corolla, anthesis, post-anthesis (Fig. 2). When microsporogenesis occurred at optimal temperature (22 °C), pollen viability gradually decreased remaining quite high along the whole flowering, ranging from 97.60% (closed corolla) to 76.77% (post-anthesis) (Fig. 2a). Conversely, higher temperature (30 °C) during microsporogenesis (flower development) drastically reduced pollen viability from 57.49% (closed corolla) to 1.32% (post-anthesis) (Fig. 2b). According to the ANOVA, the main effect of the incubation temperature was also significant (*p* < 0.01) but it was strongly influenced by the interaction with the microsporogenesis temperature. Particularly, data showed that pollen in which microsporogenesis had occurred at optimal temperature, resulted more thermotolerant to the following exposure to incubation temperature; indeed, almost all pollen grains that lactophenol-cotton blue test detected as not aborted at closed corolla stage (99.22%) remained viable till anthesis stage without significant difference between 30° incubation (91.54%) at 22 °C (95.10%) (Fig. 2a).

Higher temperature during microsporogenesis strongly reduced pollen thermotolerance to higher incubation temperature: indeed, pollen viability after 30 °C incubation resulted significantly lower than 22 °C in each phenological stage and most importantly, almost all grain did not survived 30 °C incubation at anthesis stage (6.56%) (Fig. 2b).

Overall, results showed that higher temperature occurring during microsporogenesis amplified the effect of the incubation temperature on pollen viability.

**Fig. 3:**
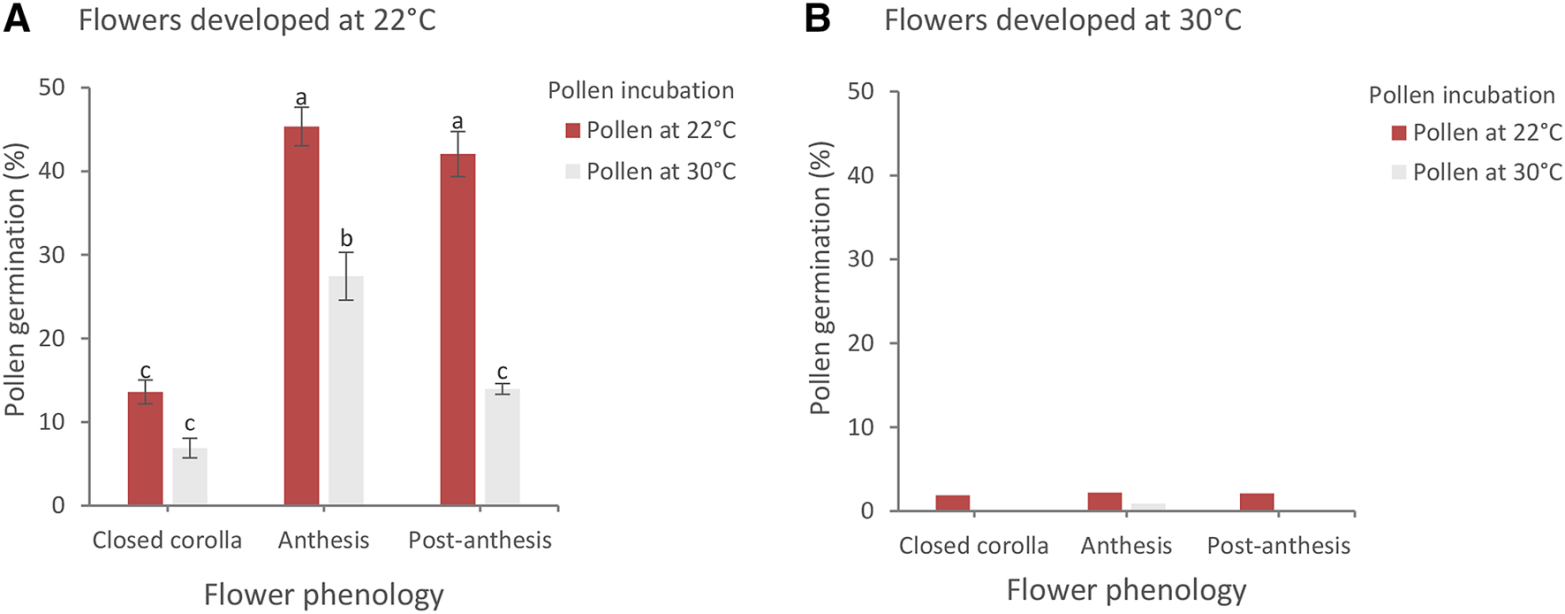
Germinability of pollen from flower at different phenological stages and incubated for 72 h at 22 °C and 30 °C. Data from flowers in which microsporogenesis occurred at 22 °C **(a)** and 30 °C **(b)**. Significant differences between phenological stages are expressed with different letters (*p* < 0.05). Bars represent ± SE

Effects on gametophyte functionality (pollen tube development) of high temperature treatment exerted during the microsporogenesis resulted significant. Data on pollen grains developed under the two temperatures and subsequently incubated to germinate at the same and at cross changed temperatures showed clear significant differences. More specifically, the three-way ANOVA revealed as significant (*p* < 0.01) the main effect of each factor and their interaction on germination percentage. According to the results, when microsporogenesis occurred at 30 °C, almost no grain developed a pollen tube independently from incubation temperature and flowering stage (Fig. 3b). When microsporogenesis occurred at optimal temperature, highest percentages of germinability occurred using pollen from flowers at the anthesis and percentages significantly decreased in pollen from flowers in post-anthesis (Fig. 3). Post-hoc analysis on the interaction between flower phenological stages and incubation temperature showed that at 22 °C the decrease in pollen germinability was not significant passing from anthesis to post-anthesis. Incubation at 30 °C temperature not only significantly decreased pollen germinability in comparison to lower temperature but halved the percentage from anthesis (27.44%) to post-anthesis stage (13.96%) (Fig. 3a).

Data on germinability of pollen from flowers at closed corolla stage deserve special considerations. Percent germination of pollen from closed-corolla flowers resulted much lower than that from flowers at the anthesis and germinability apparently increased passing from the closed corolla to the anthesis stage. Such a phenomenon is not linked to the loss of viability (that instead resulted very high using the LCB and DAB tests). In-depth microscopy observations showed that when flowers are developed at 22 °C, pollen in buds at the stage of closed corolla represent gametophytes not yet mature to develop the pollen tube (Fig. 1).

Overall, results of germination tests revealed that both microsporogenesis temperature and the following exposure to incubation temperature are essential to ensure pollen tube development at anthesis stage when pollination should naturally occur.

Further microscope observations aimed to deepen the effects of high temperature on pollen ontogenesis and to identify possible cytological differences during pollen development, revealed large differences on pollen development associated to temperature exposure of the anthers during microsporogenesis. Analysis of anther sections stained with aniline blue showed that high temperature conditions during microsporogenesis altered the timing of the developmental stages of pollen throughout flowering (Fig. 1). At the stage of closed calix, pollen mother cells, tetrads and uni-cellular microspores were observed in both treatments. Moreover, at the same stage, some microspores passed to the “vacuolated phase” only in anthers exposed to high temperature. At optimal temperature, pollen development resulted slower, and vacuolated microspores were observed from closed corolla stage on. Besides, temperature exposure of microspores affected the timing of the bi-cellular stage.

After the microsporogenesis, almost all microspores exposed to high temperature prematurely underwent the first haploid mitosis turning into mature pollen grains (bi-cellular gametophytes) at the stage of closed corolla. In flowers developed at 22 °C, the bi-cellular stage became visible only from the anthesis stage, when mature pollen is expected to be ready for dispersal. From anthesis stage on, aniline blue stain highlighted differences in viability between the temperature treatments. In flowers developed at 22 °C mature pollen was well-developed with densely stained cytoplasm from anthesis to post-anthesis stage. Differently, in anthers developed under high temperature, pollen cytoplasm drastically degenerated from closed corolla stage on and almost all pollen resulted aborted at post-anthesis.

Overall, cytological analysis allowed to clarify that the high temperature speeded up microspore transition into the male gametophyte (bi-cellular stage) and reduced pollen lifespan throughout flowering. In flowers developed at optimal temperature the presence of mature pollen in the anthers occurred as expected at the anthesis stage, while in flowers developed under high temperature pollen was ready for dispersal and fertilization when the flower was still a bud. At high temperature most of grains had lost their viability from anther dehiscence on and grains resulted too old to germinate at the anthesis stage.

## Discussion

The experimental setup and design of using reversible combination of optimal and high temperature conditions during microsporogenesis and microgametogenesis allowed to disclose that a heat stress occurring only during microspore formation may become manifest later on the male gametophyte. Data showed that the drastic reduction of the male gametophyte functionality occurred even if the number of well-developed pollen grains at anther dehiscence resulted comparable to that of pollen developed at optimal temperature. Further microscopic analyses on pollen ontogenesis explained the relations between high temperature during microsporogenesis and high reduction of the male gametophyte functionality. The investigation revealed that heat conditions accelerate the senescence processes up to the point that pollen although well-developed, is mostly dead already at anthesis, so even before pollination dispersal. These results are critical for the achievement of the reproductive success in terms of seed/fruit production, especially considering that frequency of extreme weather events is expected to increase in a climate change scenario (Hedhly 2011). In such scenarios, summer crops such as tomato, will be more often exposed to heat waves even during the earliest stages of flowering when pollen is still developing. To date, other studies have tested sensitivity of pollen to thermal insults which frequently occurs for summer crops growing both in open field and in greenhouses (e.g. Porch and Jahn 2001; Mesihovic et al. 2016; Paupière et al. 2017a).

Reproductive success and the achievement of the seed-to-seed cycle is fundamental not only for crop cultivation also to reach the ambitious objective of realising complete self‐sustaining cultivation systems for Space habitats (De Micco et al. 2014). Studies on plant biology in space reported that microgravity significantly affects pollen functionality interfering on several processes, including callose deposition at the end of the microsporogenesis (Kuang et al. 1995) or callose plugs formation in the male gametophyte (De Micco et al. 2006). Nevertheless, in addition to microgravity, interactions with other environmental factors have been identified as major contributors to male sterility in experiments conducted in space (Levinskikh et al. 2000; Campbell et al. 2001; Veselova, 2003). Sudden changes of temperatures can occur on board of space stations during the period needed to close the seed-to-seed cycle. Considering that tomato is one of the crop species selected for cultivations in space, our study furnishes new insights to define the scientific requirements for future experiments on tomato reproduction in space and to correctly interpret the results.

Focusing on the way tomato plants respond to the environmental temperature during reproduction, previous research reports high variability in pollen response within 22-30 °C temperature depending on how/when the temperature treatment was performed. For instance, Paupière et al. (2017a) reported significant loss of pollen viability on tomato when plants were exposed to high temperatures during the whole flowering. Conversely, Pham et al. (2020) did not found difference in terms of viability when pollen previously developed at optimal temperature was incubated at high temperature. Our results suggest that this variability in pollen thermo-tolerance is strictly linked to the pollen developmental stage targeted by heat treatment. Other studies on the effect of heat stress on tomato pollen report specific cytological anomalies including interferences during starch accumulation (Pressman et al. 2002) or exine formation and microspore vacuolization (Giorno et al. 2013); these processes occur during pollen ontogenesis and therefore might be involved in the lifespan and in the senescence speed of the pollen.

In the present work, pollen from flowers heat-treated during microsporogenesis always resulted significantly lower in viability compared to optimal temperature treatment while pollen in which microsporogenesis occurred at optimal temperature well tolerated both 22 °C and 30 °C incubation. Therefore, data showed that microsporogenesis occurring in the earliest stage of flower bud development represent a highly sensitive phase to high temperature exposure along the pollen ontogenesis. Although heat stress has already been reported to cause several dysfunctions in important monocot and dicot crops during early micropore stage, (Porch and Jahn 2001; Giorno et al. 2013; Jagadish et al. 2014; Szalay et al. 2019; Masoomi-Aladizgeh et al. 2020) none of these studies have described implications of these heat-stress induced defects on subsequent mature pollen viability and germinability, both essential to ensure fertilization success and crop productivity. According to our results, it seems that uni-cellular microspores are less adapted to deal with environmental factors until their transition into bi- or tri-cellular pollen grains.

Different strategies in response to heat stress have been described in pollen to preserve viability from anthesis to stigma landing including dehydration, accumulation of osmolytes and synthesis of protective molecules such as heat stress proteins enforcing membrane stability (Pacini and Dolferus 2019). Differences between uni-cellular microspores and two or three-cellular male gametophytes in tolerating high temperatures, could be related to their different capability to express their haploid genes to overcome environmental constraints. Therefore, results of the present study should be taken into account to perform successfully gametophytic selection in breeding protocols for new cultivars best adaptable to heat stress both for future space missions and for cultivations on earth.

Our data showed that the effects of high temperatures were even more severe on pollen germinability than on viability. Such a phenomenon has been reported for species other than tomato (Aronne et al. 2015; Jiang et al. 2015; Djanaguiraman et al. 2018). However, in our case a few Celsius degrees over optimal temperature occurring in the earliest stage of flowering resulted fatal for the gametophytes therefore causing a reproductive failure. Considering that pollen grains detected as viable through DAB reaction do not necessarily develop pollen tubes (Dafni 1992), it is therefore reasonable that pollen from the high temperature treatment showing a very low viability did not germinate. Germinability of pollen developed at optimal temperature increased along flowering and reached the maximum percentage at anthesis. These results are in line with our cytological analysis in which we assessed that the highest germination percentage corresponds to the bi-cellular stage that in the optimal temperature treatment occurred at anthesis. Indeed, tomato pollen is generally dispersed as bi-cellular gametophytes and the second mitotic division only occurs after germination on the stigma. In the high temperature treatment, all the developmental stages resulted speeded up compared to optimal temperature treatment. In particular, the transition into the bi-cellular stage representing the condition of mature pollen had already occurred before anthesis. We thus highlight that high temperatures during microsporogenesis also causes a premature transition of microspores into bi-cellular pollen to ensure mature pollen formation ready for dispersal but reducing pollen lifespan throughout flowering. Therefore, both direct effects of high temperature on the earliest stages of flower bud development and reduction of pollen lifespan throughout flowering can be responsible for a drastic loss in pollen viability and germinability.

Finally, the dwarf variety of tomato used for our experiment proved to be effective and further usable as a model for more studies on pollen functionality allowing to grow plants in small chambers where environmental parameters can be finely modulated and different temperature treatments can be performed during specific pollen developmental stages.

In conclusion, we successfully used tomato ‘Micro-Tom’ as model plant to contribute to the studies on the effects of heat stress on reproduction and verified the hypothesis that high temperature occurring during microsporogenesis can affect the subsequent functionality of the male gametophyte. Results also allowed to disclose that short periods of high temperature (as during the occurrence of heat waves) can accelerate pollen senescence processes priming a fatal shortening of the gametophyte lifespan and consequently a drop-off to zero of the viability before pollen is transferred by pollination.

### Author contribution statement

MI and GA equally contributed to conceptualization, investigation and writing.

## Data Availability

(Data transparency) Raw data can be provided upon request.

## Code availability

(software application or custom code) Not applicable.
